# Duke Surgery Patient Safety: an open-source application for anonymous reporting of adverse and near-miss surgical events

**DOI:** 10.1186/1750-1164-1-5

**Published:** 2007-05-01

**Authors:** Ricardo Pietrobon, Raquel Lima, Anand Shah, Danny O Jacobs, Matthew Harker, Mariana McCready, Henrique Martins, William Richardson

**Affiliations:** 1 Center for Excellence in Surgical Outcomes, Department of Surgery, Duke University Medical Center, DUMC 3094, Durham, NC 27710, USA; 2 Department of Surgery, Duke University Medical Center, DUMC 3704, Durham, NC 27710, USA; 3 Division of Orthopaedic Surgery, Department of Surgery, Duke University Medical Center, DUMC 3077, Durham, NC 27710, USA

## Abstract

**Background:**

Studies have shown that 4% of hospitalized patients suffer from an adverse event caused by the medical treatment administered. Some institutions have created systems to encourage medical workers to report these adverse events. However, these systems often prove to be inadequate and/or ineffective for reviewing the data collected and improving the outcomes in patient safety.

**Objective:**

To describe the Web-application Duke Surgery Patient Safety, designed for the anonymous reporting of adverse and near-miss events as well as scheduled reporting to surgeons and hospital administration.

**Software architecture:**

DSPS was developed primarily using Java language running on a Tomcat server and with MySQL database as its backend.

**Results:**

Formal and field usability tests were used to aid in development of DSPS. Extensive experience with DSPS at our institution indicate that DSPS is easy to learn and use, has good speed, provides needed functionality, and is well received by both adverse-event reporters and administrators.

**Discussion:**

This is the first description of an open-source application for reporting patient safety, which allows the distribution of the application to other institutions in addition for its ability to adapt to the needs of different departments. DSPS provides a mechanism for anonymous reporting of adverse events and helps to administer Patient Safety initiatives.

**Conclusion:**

The modifiable framework of DSPS allows adherence to evolving national data standards. The open-source design of DSPS permits surgical departments with existing reporting mechanisms to integrate them with DSPS. The DSPS application is distributed under the GNU General Public License.

## Background

### Anonymous reporting of adverse and near-miss events

Near miss events occur in every medical facility all over the world. Studies have shown that 4% of hospitalized patients suffer from an adverse event caused by the medical treatment administered [1,2]. These mistakes can result in patient discomfort, irreversible injury, or even death. Although patient safety is a topic of major emphasis for medical facilities, efforts to investigate the causes and prevention of these errors have been insufficient, reflecting a general lack of awareness of the problem [2]. As a consequence of this oversight, the need for effectively collecting and understanding data about these adverse events is paramount in improving patient safety. Some institutions have created systems to encourage medical workers to report these adverse events so that they may be formally documented and reviewed. However, these systems have often proven to be inadequate or ineffective for reviewing the data collected and improving the outcomes in patient safety. Studies on the handling of incident reports indicated that no clear and consistent methodology across a number of organizations were of an appropriate added value to patient safety mechanisms [1].

### Existing approaches

Although systems have been created for reporting adverse events, there does not exist, to the best of our knowledge, an open source application for reporting patient safety. The open source application is a software system that permits the usage and modification of its source code. In laymen's terms, "open-source," by definition, implies that the software allows anyone to improve, redistribute, and share it with others [3-8]. There are a number of Web-based event reporting systems in health care. US Pharmacopeia's MedMarx system [9] and the Institute for Safe Medication Practices' Medication Errors Reporting Program [10] collect data on medication errors. The Emergency Care Research Institute (ECRI) [11] collects reports of adverse events involving medical products. Mekhijian et al recently implemented a voluntary reporting system that collects data about errors, events, and near misses solely at Ohio State University. All of these systems focus on a specific type of event (e.g., medications) or are localized at one institution. While virtually all medical institutions have some in-hospital mechanism for reporting incidents, typically these systems are mandatory and non-anonymous. This allows blame to be focused on individuals, and, as a result, these systems result in grave underreporting.

A single-institution studyfound that a significant increase in reporting occurred within the first year of a hospital-wide implementation of a Web-based electronic safety event reporting system [12]. Twenty-two percent of the safety events reported resulted in patient harm; 16% of these were near misses. Nurses entered 73% of the events and physicians only 2%. Miscoding of event details and underreporting were common. Most of the events were reported by nurses [12] which highlights the need of a system that allows all staff, including physicians and other team members, involved with patient care to report incidents. At the same time, more than one report may be submitted for the same episode and the system must be prepared to identify such cases. These results suggest that more work is needed to involve care team members in reporting, improve the accuracy of the submitted information, and prioritize and streamline event analysis. Few national surveillance systems [13,14] and other institutional safety reporting systems value the voluntary, anonymous, and confidential aspects of collecting information about adverse events and near misses. Some of these provide feedback in the form of monthly reports, case discussions and/or newsletters [15], rather recognizing the importance of educational interventions such as safety awareness and learning how to use the reporting system [16,12]. Such features are believed to result in great improvement for the reporting systems.

### Objective

We describe an open-source Web application, Duke Surgery Patient Safety (DSPS), designed at our institution for the anonymous reporting of adverse and near-miss events as well as regular reporting to physicians and hospital administration. This program was designed to streamline this process in the Department of Surgery at our institution; nonetheless, it can be adapted to any patient care department. The primary goal was to design a program that would streamline adverse-event reporting with minimal time required by reporters in the ever-busy and high volume surgical environment. To the best of our knowledge, this is the first ever open-source program created to streamline the reporting of adverse events. More importantly, this program can be freely modified to sync with the existing reporting systems of institutions.

## Implementation

### Goals

The primary goal of the DSPS application is to provide a mechanism for anonymous reporting of adverse events. In addition, DSPS should also automate repetitive tasks associated with the administration of patient safety initiatives, such as (1) send regular personalized reminders by e-mail to all potential reporters, (2) allow administrators to properly store, make changes, and reject individual reports as deemed necessary, and (3) generate automated periodic reports for individual practitioners and hospital administrators summarizing adverse report rates in a graphical format.

### Design objectives

The overall objective of the DSPS project was to build a Web-based application that would allow for the anonymous reporting of adverse events. Before designing the application, we analyzed similar tools, including commercially available products, adverse event reporting systems from academic institutions, and recommendations from the Institute of Medicine [17].

Our search resulted in a series of technical requirements that are highly desirable for the application:

• User interface should be simple and straightforwardto enhance reporter participation

• The application should run on an Intranet to facilitate access by multiple computers within the same institution

• Reporters should have a minimum set of categories to classify their complications according to a standardized coding system determined by the institution, followed by a unique free-text description of the adverse or near-miss event (ANME) and its potential cause

• Reporters should be allowed to describe more than one ANME per patient without having to make a second patient entry

• Administrators should have the ability to change ANME that are inaccurate and reject others that are not considered to be adverse events

• Administrators should have the ability to check the ANME before it is accepted as a true event

• In order to incentives reporting in the surgical setting, the application should remind reporters about its existence on a regular basis, also sending them reports about the numbers of complications submitted by their division

• Data extraction should be easy, allowing administrators to perform further analyses using external tools

The results from the search were implemented in the design of our application thus accomplishing the following design objectives:

• Simplifying the reporting process for frontline staff

• Eliminating multiple forms used to report adverse events

• Increasing the quantity and quality of occurrence data

• Improving response time by linking reports to department leadership and key personnel (clinical nurse specialist, unit educator)

• Improving evaluation and follow-up through a structured framework

• Enhancing the quality and safety of patient care and the employee work

DSPS should ideally adhere to national data standards. Although ideal, achieving consensus on such standards remains a controversial matter. A recent effort by the National Surgical Quality Improvement Program [18] has attempted to classify some of the most common postoperative adverse events (Table 1). However, currently this classification is not comprehensive for most surgical subspecialties and, therefore, we have attempted to design a method that will accomplish the following goals:

**Table 1:** List of adverse events as listed in the National Surgical Quality Improvement Program (NSQIP), 2005

Wound Ocurrences
Superficial Incisional SSI
Deep Incisional SSI
Organ/Space SSI
Wound Disruption
Other (ICD-9)
Respiratory
Pneumonia
Unplanned Intubation
Pulmonary Embolism
On Ventilator > 48 hours
Other (ICD-9)
Urinary Tract Occurrences
Progressive Renal Insuficiency
Acute Renal Failure
Urinary Tract Infection
Other (ICD-9)
CNS Occurences
Stroke/CVA
Coma >24 hours
Peripheral Nerve Injury
Other (ICD-9)
Cardiac Ocurrences
Cardiac Arrest req. CPR
Myocardial Infarction
Other (ICD-9)
Other Ocurrences
Bleeding > e units RBCs
Graft/Prosthesis/Flap Failure
DVT/Throbophebitis
Systemic Sepsis
SIRS
Sepsis
Septic Shock
Other (ICD-9)

• Allow for data exchange across different surgical services and institutions

• Be flexible to ensure that specific needs of each surgical division are met

### Software architecture

**Figure 1:**
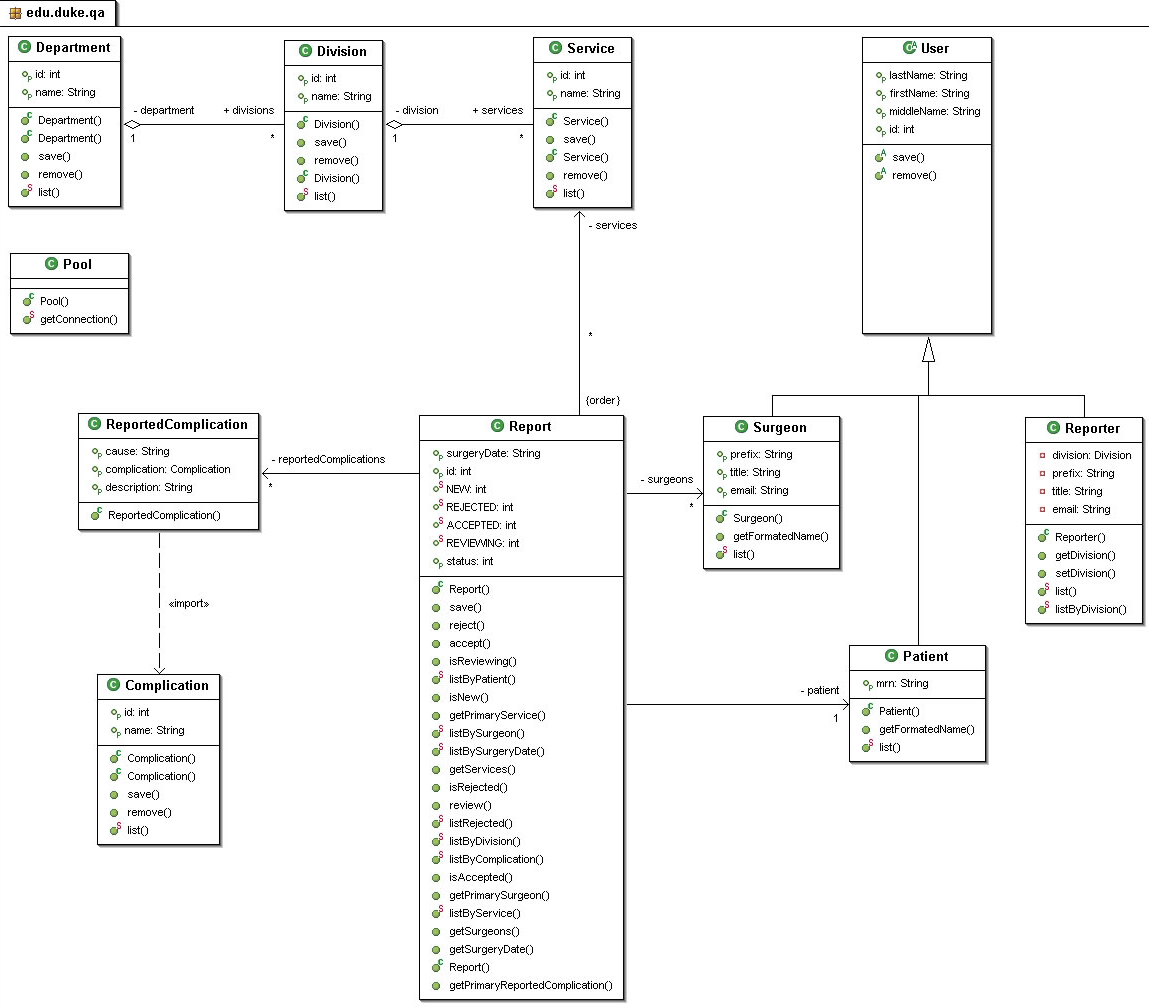
Model-View-Controller (MVC) as its design model.

**Figure 2:**
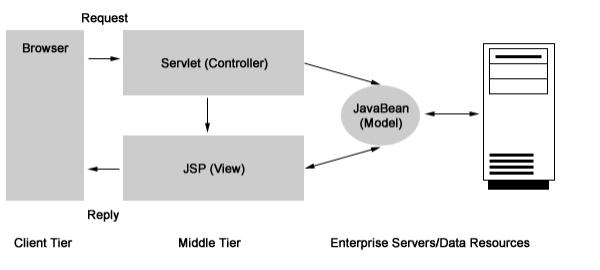
Controllers of the Model.

**Figure 3:**
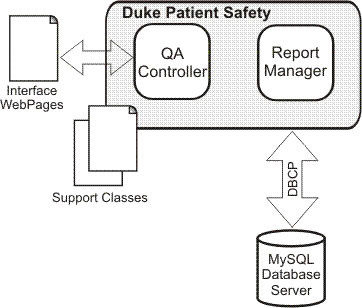
DSPS architecture.

The software architecture was developed using a model similar to previous software applications from our group [19-21]. The programming language used was Java and the design model was Model-View-Controller (MVC) (Figure 1). MVC's three components are: a central Model, Views, and Controllers of the Model (Figure 2). The Views and the Controller are the application's interface. They represent the Model to the user. As for the central Model, it displays the logic, as well as the database access, numeric algorithms, and algorithms for data manipulation. The Controller provides information to the Model while the View passes it from the Model to the users. Figure 3 is a diagram of the DSPS architecture.

### Software interface

The Duke Surgery Patient Safety application provides interfaces for three classes of users: ANMEReporters, Administrators, and Report Receivers.

### Activity flow

**Figure 4:**
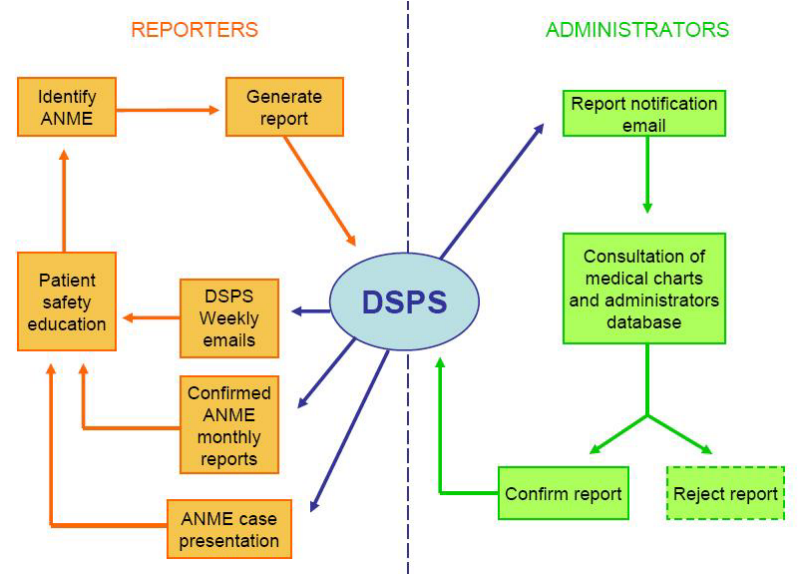
flow of activities in the software architecture.

Before the application was built, we evaluated the usual flow of activities for ANME reporting and how the application would be integrated into this system. This analysis resulted in the following flow of activities in the software architecture (Figure 4):

• Weekly e-mails are directly sent to health care professionals working with patients reminding them about the importance of ANME reporting and reminding them of the central Web site.

• When an ANME is identified, health care professionals go to the Web site and generate the report using a single reporting interface.

• Administrators receive an e-mail soon after a new ANME is reported. They access DSPS and receive the information that will allow them to investigate the ANME in further detail. This checking is performed through consultation of medical charts and administrative databases.

• Once the ANME is confirmed, the administrator returns to DSPS and confirms its existence. Otherwise, the report can reject by the administrator.

• Confirmed ANME are included in a monthly report with information customized to individual practitioners and divisions.

### ANME reporters

**Figure 5:**
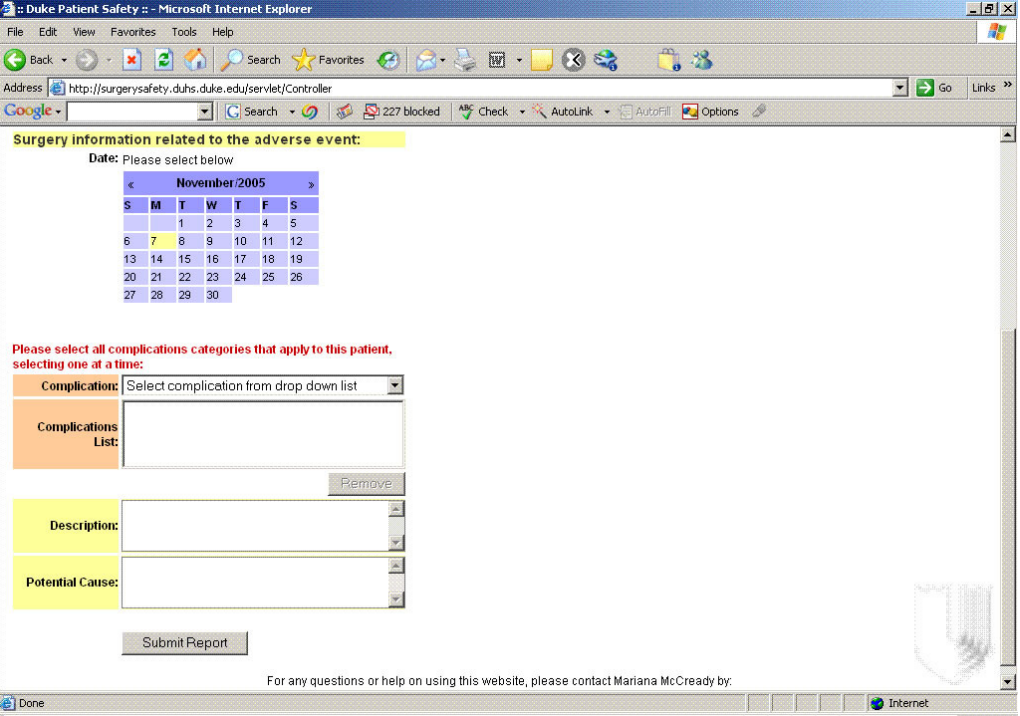
Complication Report.

The interface for ANME reporters includes functionality that allows reporters to perform the following functions:

• Choose the surgical division, subspecialty, and primary surgeon and/or house staffassociated with the ANME.

• Provide information that will allow for identification of the patient for whom the ANME is being reported.

• Select all pre-defined complication categories that are applicable to this patient, including a text description where reporters can add additional information as needed, including potential cause. Reporters also have the ability to delete complications prior to submission if a category is erroneously selected (Figure 5).

• Since at our institution the most frequent users of the system are surgical residents, the reporter interface also has a link to a repository of previous patient safety presentations. Usually residents prepare their presentations based on previous ones, andthe inclusion of this additional resource ensures that they will frequently come to the DSPS site, thus serving as a constant reminder of the importance of patient safety reporting.

### Report receivers and administrators

**Figure 6:**
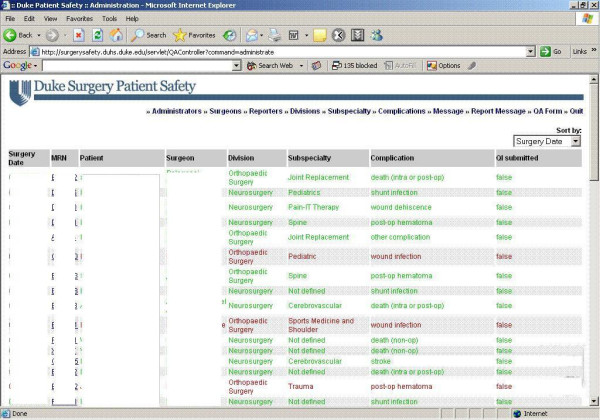
Report Receivers and Administrators.

The administrative interface was designed to allow the administrator complete control over all existing tasks (Figure 6). These include:

• Receive e-mail when ANME report is made

• Check reliability of ANME report and either accept or reject it

• Add new administrators. This function is of exclusive use of departmental administrators, so that they can delegate the function of divisional representatives. The administrator can also add new divisions and respective subspecialties, providers, reporters

• Add and modify new sets of complication categories that are specific to divisions

• Export ANME data in a spreadsheet so that it can be manipulated using data analysis software (e.g., text mining, statistical packages, neural networks, etc)

• Define report format and respective release dates for providers.

• Generate messages sent to all potential reporters (health care professionals working with patients) on a regular basis. These messages usually contain a message stating the importance of ANME reporting, also providing links to DSPS.

• Add educational files to an educational repository. These files function as a library aiding the resident who, at our institution, is in charge of the monthly patient safety case presentation.

### Usability evaluation

Comprehensive usability tests were performed by formal usability analysis and a field observation period. Formal usability analysis was conducted in a manner similar to a previous study by the authors' institute [20] In short, twelve physician scientists (n = 10) and nurses (n = 2), from two institutions, were asked to participate to avoid bias from authors participating in the development of the DSPS application. Six users tested the reporter's interface and the other six tested the administrator's interface. Each of these users, by self-report, rated as having average computer literacy with no previous experience with the DSPS application. Formal usability tests followed a protocol where users were observed by two evaluators (who were available to answer any questions from users) and had to complete assigned regulatory tasks. The following DSPS application factors were evaluated: speed, easy of learning, easy of using, understanding of functionality, and navigation. The evaluators who observed the users made detailed notes about number of errors made while using the application. Each participant was instructed to answer a questionnaire at the end of the formal usability analysis with items about interface problems, missing features, and suggestions for overall improvement.

As with previous studies by these authors [20], field observations were conducted by one of the co-authors (MM) and consisted of observation of researchers and research administrators during the execution of common regulatory activities using DSPS. Meetings were held with investigators and research administrators in order to obtain detailed feedback concerning any concerns or suggestions for improvement, however we acknowledge that further studies with more users are needed for long-term testing.

## Results

### Implementation

The current version of DSPS was designed to allow for the anonymous reporting of adverse events, their evaluation and follow-up, while reducing the number of forms used and making the reporting process easier leading to an improvement in the quality and safety of patient care. Its current design incorporates the numerous suggestions received from formal and field usability tests. The DSPS application has been implemented in the Department of Surgery atDuke University Medical Center.

When making an anonymous report, the reporters are asked to enter the division/department name, surgeon and, if appropriate, subspecialty related to the adverse event by choosing from drop down boxes available. Then, they need to provide patient information such as MRN, last name, first name and middle initial. Next, reporters select the date of the complication in a calendar. All complication categories that apply to the specific patient are selected, selecting one at a time from a drop down list. In sequence, reporters can add additional information about the adverse event descriptions and potential cause by typing into the appropriate text areas. At last, they mark the severity of complication based on Complications Grading System:

• Grade I – Any deviation from the normal postoperative course without the need for pharmacological treatment or surgical, endoscopic, and radiological interventions. Allowed therapeutic regimens are: drugs as antiemetics, antipyretics, analgesics, diuretics, electrolytes, and physiotherapy. This grade also includes wound infections opened at the bedside.

• Grade II – Requiring pharmacological treatment with drugs other than those allowed for grade I complications. Blood transfusions and total parenteral nutrition are also included.

• Grade III – Requiring surgical, endoscopic or radiological intervention.

• Grade IV – Life-threatening complication (including CNS complications) requiring IC/ICU management.

• Grade V – Death of a patient.

When the reporters are done, they must click on the "submit report" button. The department administrator will be notified immediately. Each administrator will check the consistency of reports made in their departments and either accept or reject them. Although the adverse event reports are screened by the administrator, one should be aware that researchers, physicians and national organizations interested in patient safety may have different goals.

### Data standards

In DSPS, our approach has been to present surgical specialties with existing standards that are internal to each of the surgical departments using the application. Further modifications are made in the attempt to make the variables compliant with what exists in other divisions, ultimately allowing comparisons across subspecialties.

### Usability

#### 1. Formal usability

Results from formal usability testing are summarized in Table 2. Formal usability results demonstrated that, from the perspective of researchers, the DSPS application presented excellent speed (11/12 users strongly agreed), was easy to learn and use (10/12 users strongly agreed), had a functionality that was easily understandable (10/12 users strongly agreed), and a navigation that was intuitive (12/12 users strongly agreed). No users reported any problems in the questionnaire that was presented after formal usability testing.

**Table 2:** Formal usability

DSPS speed is excellent.	Strongly disagree	0/12
	Disagree	0/12
	Neutral	0/12
	Agree	1/12
	Strongly agree	11/12
DSPS is extremely easy to learn	Strongly disagree	0/12
	Disagree	0/12
	Neutral	1/12
	Agree	1/12
	Strongly agree	10/12
DSPS is extremely easy to use	Strongly disagree	0/12
	Disagree	0/12
	Neutral	0/12
	Agree	4/12
	Strongly agree	8/12
It is very easy to understand all functionality available within DSPS (e.g., download files, upload files, etc)	Strongly disagree	0/12
	Disagree	0/12
	Neutral	1/12
	Agree	1/12
	Strongly agree	10/12
The navigation in DSPS is highly intuitive	Strongly disagree	0/12
	Disagree	0/12
	Neutral	0/12
	Agree	0/12
	Strongly agree	12/12

#### 2. Field usability

The first two months of field usability measurement were primarily focused on fixing software bugs, namely non-functional links to other Web pages and click buttons that did not work. DSPS users were encouraged during this time to submit their comments and suggestions directly to one of the co-authors (MM) who investigated any issues with the programmer (HM).

During this phase, one issue that was corrected was the automatic email that is sent on a weekly basis to all surgeons and reporters who use DSPS for reporting. Initially, it was not being received by the users on certain weeks. Upon detection of the error, the programmers were able to readily correct the issue. Also during this phase, multiple users identified that the link they were receiving in the weekly automatic email was not working correctly. The users would click on the link and get an error message. However, upon reviewing this issue, it was an error attributable to the email system used at our institution, which frequently can not open links included in an email. To remedy this situation, additional instruction was included in the weekly email to copy and paste the link into a browser window.

## Discussion

To our knowledge, this is the first description of an open-source application for reporting patient safety related events. DSPS provides a mechanism for anonymous reporting of adverse events and helps the administration of patient safety initiatives. At the time of this article, Duke Surgery Patient Safety has only been used within a few departments at Duke University, including the Department of Surgery; however, the free distribution of its source code under the GNU Public License is expected to spread its use among other departments and colleague institutions. Although this is possible, we have not tested the cross-application of our software yet, and therefore, we are unable to discuss possible difficulties that might arise. We recommend that potential users consult their institutional IT department for guidance in installation and customization.

There are many web-based reporting systems available, but most of them are restricted to one institution or they collect data on just one type of event such as medication errors and adverse events involving medical products. An example is a passive reporting system for adverse effects caused by vaccine. The events are voluntarily reported by the person who experiences them which leads to great underreporting, unconfirmed diagnoses and lack of temporal association [14].

Much of the difficulty with underreporting stems from the fact that many reporting systems focus blame on individuals rather than on the prevention of similar events. The importance of developing consciousness and awareness of patient safety, rather than generating individual blame, has the potential to greatly enhance the patient safety reporting system and the incentive to utilize the system. Ultimately, such system has the potential to improve patient morbidity and mortality in departments where administrative time is limited for members of the patient care team. We are confident that the simplicity and streamlined reporting of DSPS make it a valuable asset for our institution.

## Conclusion

### Current utilization

DSPS is currently used by the Divisions of Orthopaedic Surgery, Neurosurgery, General Surgery, OHN (Otolaryngology, Head and Neck), Pediatric Surgery, Plastic Surgery, Urology and Cardiothoracic Surgery at Duke University Medical Center (DUMC). It is currently being expanded to other divisions within the Department of Surgery at DUMC, as well as other academic and non-academic centers.

### Potential uses

Although DSPS was originally developed to fit the patient safety needs of the Department of Surgery at DUMC, its modification for use at other institutions is simple and can be easily accomplished by a few modifications in the open-source application. Several features are planned for future versions of DSPS. First, integration with administrative databases would enable our group to flag adverse events in a more automated manner. For example, if hypoglycemia is detected in an inpatient, this might indicate an adverse event (mismanagement of insulin treatment). Second, future versions should have the ability to obtain denominators (total number of cases) from administrative databases in order to generate ANME rates. These rates would be more meaningful that simple frequencies. Implementations of both features require integration of administrative databases with heterogeneous architectures, therefore requiring an interface that may integrate well with XML standards as well as possibly legacy systems.

### Potential reactions

According to the Committee on Data Standards for Patient Safety of the Institute of Medicine [22], three main reactions corresponding to the "cycle of fear" can occur once safety data are released. First, "kill the messenger" reactions are triggered and there is an attempt to shift the blame by questioning the measurement system and those conducting the evaluation. Second, "filter the data" starts as it is much easier to achieve better scores by 'gaming the system' than by actually changing and upgrading procedures. Third, "micromanage" response takes place. Reacting to immediate variation in the process data instead of conscientious investigation and remodeling will make the system less efficient. It is the hope of the authors that the release of patient safety reports through DSPS promotes the development of positive learning systems. Education attempts to change the shape of the performance distribution by improving all parts of the process [23]. However, to accomplish this learning system phase, a self-reporting application such as DSPS should not be an isolated solution. A crucial resolution is to give emphasis to safety itself rather than blaming. For example, we hope that ANME case discussions provide opportunity for improvement.

## Availability and requirements

• Project name: Duke Surgery Patient Safety (DSPS)

• Project home page:  (click link for Free software)

• Operating system(s): Linux/Windows

• Programming language: Java

• Other requirements: Java 1.3.1 or higher, Tomcat 5.x or higher

• License: GNU General Public License

• Any restrictions to use by non-academics: none

## Abbreviations

• DSPS-Duke Surgery Patient Safety

• ANME-Adverse and near-miss events

• DUMC-Duke University Medical Center

## Competing interests

The author(s) declare that they have no competing interests.

## Authors' contributions

All authors have read and approved the final manuscript.

• RP – designed the application, assisted with software testing, conducted the usability tests, and wrote the first draft of the manuscript

• RL – assisted with the design of the application, reviewed the manuscript for intellectual content

• AS – assisted with the design of the application, reviewed the manuscript for intellectual content

• DOJ – assisted with the design of the application, reviewed the manuscript for intellectual content

• MH – assisted with the design of the application, reviewed the manuscript for intellectual content

• MM – assisted with the design of the application, assisted with field testing and software debugging, reviewed the manuscript for intellectual content

• HM – assisted with the design of the application, wrote and tested the source code for the application, reviewed the manuscript for intellectual content

• AM – assisted with the design of the application, assisted with field testing, reviewed the manuscript for intellectual content

• WR – assisted with the design of the application, assisted with field testing, reviewed the manuscript for intellectual content

## Appendix

### Programming language and development methodology

DSPS was developed primarily using JAVA programming language, operating on a Tomcat server and having a MySQL database as its backend. We used a prototyping development methodology, starting with paper prototype and drawing requirements from that prototype. Once the requirements were reviewed, a first version was developed and tested. This cycle was repeated until the currentversion was obtained.
